# Associations of the Disrupted Functional Brain Network and Cognitive Function in End-Stage Renal Disease Patients on Maintenance Hemodialysis: A Graph Theory-Based Study of Resting-State Functional Magnetic Resonance Imaging

**DOI:** 10.3389/fnhum.2021.716719

**Published:** 2021-12-13

**Authors:** Die Zhang, Yingying Chen, Hua Wu, Lin Lin, Qing Xie, Chen Chen, Li Jing, Jianlin Wu

**Affiliations:** ^1^Department of Radiology, Affiliated Zhongshan Hospital of Dalian University, Dalian, China; ^2^Department of Radiology, Shenzhen Third People’s Hospital, Shenzhen, China; ^3^Department of Radiology, National Cancer Center, National Clinical Research Center for Cancer, Cancer Hospital, Shenzhen Hospital, Chinese Academy of Medical Sciences, Peking Union Medical College, Shenzhen, China; ^4^Department of Nephrology, Affiliated Zhongshan Hospital of Dalian University, Dalian, China

**Keywords:** end-stage renal disease, maintenance hemodialysis, graph theory, resting-state functional magnetic resonance imaging (rs-fMRI), cognitive impairment

## Abstract

**Objective:** Cognitive impairment (CI) is a common neurological complication in patients with end-stage renal disease undergoing maintenance hemodialysis (MHD). Brain network analysis based on graph theory is a promising tool for studying CI. Therefore, the purpose of this study was to analyze the changes of functional brain networks in patients on MHD with and without CI by using graph theory and further explore the underlying neuropathological mechanism of CI in these patients.

**Methods:** A total of 39 patients on MHD (19 cases with CI and 20 without) and 25 healthy controls (HCs) matched for age, sex, and years of education were enrolled in the study. Resting-state functional magnetic resonance imaging (rs-fMRI) and T1-weighted high-resolution anatomical data were obtained, and functional brain networks for each subject were constructed. The brain network parameters at the global and regional levels were calculated, and a one-way analysis of covariance was used to compare the differences across the three groups. The associations between the changed graph-theory parameters and cognitive function scores in patients on MHD were evaluated using Spearman correlation analysis.

**Results:** Compared with HCs, the global parameters [sigma, gamma, and local efficiency (Eloc)] in both patient groups decreased significantly (*p* < 0.05, Bonferroni corrected). The clustering coefficient (Cp) in patients with CI was significantly lower than that in the other two groups (*p* < 0.05, Bonferroni corrected). The regional parameters were significantly lower in the right superior frontal gyrus, dorsolateral (SFGdor) and gyrus rectus (REC) of patients with CI than those of patients without CI; however the nodal local efficiency in the left amygdala was significantly increased (all *p* < 0.05, Bonferroni corrected). The global Cp and regional parameters in the three brain regions (right SFGdor, REC, and left amygdala) were significantly correlated with the cognitive function scores (all FDR *q* < 0.05).

**Conclusion:** This study confirmed that the topology of the functional brain network was disrupted in patients on MHD with and without CI and the disruption of brain network was more severe in patients with CI. The abnormal brain network parameters are closely related to cognitive function in patients on MHD.

## Introduction

End-stage renal disease (ESRD) is defined as chronic kidney disease with a glomerular filtration rate below 15 ml/min//1.73 m^2^ or requiring permanent renal replacement therapy (Brouns and De Deyn, 2004). Maintenance hemodialysis (MHD) is one of the most important treatment methods.

Patients with ESRD are often affected by various neurological complications, such as cognitive impairment (CI). CI affects quality of life and mortality in patients with ESRD (Murray et al., 2006; Sarnak et al., 2013; Van Zwieten et al., 2019). In particular, patients with ESRD undergoing hemodialysis suffer from more severe CI (Murray et al., 2006). Moreover, a large number of unrecognized cognitive impairments in patients on hemodialysis have a significant impact on nutritional status, compliance with medical treatment, and hospitalization (Sehgal et al., 1997; Murray, 2008; Kwan et al., 2020). Therefore, exploration of the mechanism of hemodialysis-related cognitive decline in patients with ESRD is of great significance.

Neuroimaging is an important tool for studying cognitive decline in patients with ESRD (Kong et al., 2014; Chen et al., 2015; Zhang et al., 2015; Luo et al., 2016; Lu et al., 2019). These studies usually focus on changes in brain function and structure. Abnormal brain activity in regions related to cognitive function was found using resting-state functional magnetic resonance imaging (rs-fMRI) (Chen et al., 2015; Luo et al., 2016). Another study showed that altered functional connectivity of the frontal lobe is related to cognitive decline (Lu et al., 2019). Further, structural damage to the white matter is also associated with the performance of cognitive function (Kong et al., 2014; Zhang et al., 2015).

The human brain is a complex network system that can efficiently integrate and transfer information. Recently, a graph theory-based method revealed that the functional brain network in patients with ESRD was damaged, which may affect the cognitive function of patients (Park et al., 2020; Wu et al., 2020). However, the difference in brain network architecture between patients on MHD with and without CI remains unclear.

Therefore, in this study, we hypothesized that although the topology of functional brain networks in patients on MHD changes, these changes are not consistent between those with and without CI. To test our hypothesis, we used graph theory-based methods of brain network analysis to detect potential differences in the topology of functional brain networks between healthy controls (HCs) and patients on MHD with and without CI, and to explore the relationship between these possible alterations and cognitive function.

## Materials and Methods

The ethics committee of our hospital approved this study, and informed consent was obtained from all the subjects.

### Subjects

Between December 2016 and October 2018, 39 patients with ESRD on regular hemodialysis treatment (dialysis duration ≥ 3 months and 3–4 times a week) in our hospital’s hemodialysis center were enrolled in this study. The etiological factors of ESRD included: hypertension (12), chronic glomerulonephritis (10), diabetes (9), nephrotic syndrome (1), purpura nephritis (1), and unknown causes (6). The patients were divided into two groups: patients with CI [Montreal Cognitive Assessment Scale (MoCA) scores < 26] and those without CI (MoCA scores ≥ 26). Clinical data including age, sex, years of education, body mass index (BMI), and dialysis duration were collected from the electronic medical record system. During this cohort period, 25 healthy controls (HCs) who had no kidney disease, diabetes, hypertension, or other neuropsychiatric diseases were recruited from the local community.

All subjects were right-handed and the exclusion criteria were as follows: (1) age < 18 or >70 years; (2) obvious lesions (>1.0 cm) in the brain, such as cerebral infarction and brain tumor, confirmed by clinical history and conventional MRI; (3) severe heart failure and liver disease; (4) a history of drug or alcohol abuse; (5) contraindications to MRI scanning and inability to complete the neuropsychological test; and (6) translational motion greater than 2.5 mm, rotation greater than 2.5°, and mean framewise displacement values greater than 0.5 in data processing (Power et al., 2012).

### Blood Biochemistry and Neuropsychological Test

Blood samples for biochemistry tests were collected up to 3 days before MRI scanning and results for blood urea, serum creatinine, uric acid, potassium (K), calcium (Ca), phosphorus (P), red blood cell (RBC), hemoglobin (HGB), and hematocrit (HCT) were checked. Blood biochemical parameters were not tested in the HCs.

To effectively perform the neuropsychological assessments for each subject, a neuropsychological test was conducted in a relatively quiet and comfortable environment and attempts were made to ensure that the results are not affected by the subjects’ uncoordinated behavior or emotional conflict. The test was commenced within one hour of performing the MRI scan. Three scales, including the MoCA (Drew et al., 2020), Trail Making Test A (TMT-A) (Llinàs-Reglà et al., 2017), and Digit Span Task (DST) (Anil et al., 2003), were administered to assess global cognitive function, executive function, and short-term memory function, respectively. Anxiety and depression were assessed using the Zung’s Self-rating Depression Scale (SDS) and Zung’s Self-rating Anxiety Scale (SAS), respectively.

### Magnetic Resonance Imaging Data Acquisition

A 3.0-T MRI scanner equipped with a 12-channel head coil (Siemens, Verio, Germany) was used to obtain the imaging data. The participants were asked to lie quietly on the examination bed and the head was fixed with a foam sponge to reduce the impact of head movement. The scan sequences and parameters were as follows: (1) T2 fluid-attenuated inversion-recovery sequence (T2 FLAIR): repetition time (TR) = 4000 ms; echo time (TE) = 77 ms; flip angle (FA) = 150°; field of view (FOV) = 250 mm × 226 mm; slices = 20; and slice thickness = 5.0 mm. (2) T1-weighted three-dimensional (T1-3D) high-resolution: TR = 2530 ms; TE, 2.22 ms; FA, 7°; FOV, 224 × 224 mm; slices = 192; slice thickness = 1 mm. T2-FLAIR and T1-3D high-resolution images can be used to detect severe structural brain lesions (e.g., severe white matter lesions and tumors). (3) fMRI: TR = 2000 ms; TE = 30 ms; FG = 90°; FOV = 224 × 224 mm; slices = 31; slice thickness = 3.5 mm; 240 volumes were obtained for each participant. Before the scan of the functional series, all subjects were asked to remain still with their eyes closed and keep their mind clear without thinking about anything.

### Data Preprocessing

Magnetic resonance imaging image processing tools (Data Processing & Analysis for Brain Imaging toolbox^1^ (Yan et al., 2016) based on MATLAB 2013b (The MathWorks, Inc., Natick, MA, United States)^2^ software were used to preprocess the MRI data. The preprocessing steps were as follows: (1) removal of the first 10 volumes; (2) slice timing and realignment of the remaining 230 volumes; (3) regression of nuisance covariates including white matter signal, cerebral spinal fluid signal, Friston-24 parameters of head motions, and linear detrend; (4) spatial normalization using the DARTEL algorithm (Chen et al., 2020); and (5) rmoval of temporal filtering (0.01–0.08 Hz).

### Network Construction and Threshold Selection

The functional connection matrix for each subject was constructed using the GRETNA 2.0 package^3^ (Wang et al., 2015) in MATLAB 2013b. First, the whole brain was parcellated into 90 regions (nodes) based on automated anatomical labeling (AAL) atlas, which contains cortical and subcortical regions (Tzourio-Mazoyer et al., 2002). Next, the mean time series for each node was obtained, and the partial correlation coefficients of the mean time series between each node pair were calculated. Finally, a 90 × 90 functional connectivity matrix was acquired for each subject, and this matrix was converted into a binary matrix (i.e., adjacency matrices) based on a predefined threshold (described below), where the entry *a*_*ij*_ was 1, if the absolute value of the partial correlation coefficients between nodes *i* and *j* was greater than the predefined threshold. Otherwise, *a*_*ij*_ was 0.

Currently, there is no optimal or single strategy for threshold selection. Thus, a wide range of sparsity (S) was employed instead of the choice of a single threshold to meet a small-worldness as well as to perform sparse properties for each functional network (Rubinov and Sporns, 2010; Zhang et al., 2011). According to previous studies (Wu et al., 2020), the wide sparsity for brain network construction in this study ranges from 0.1 to 0.4, with an interval of 0.01.

### Network Parameters

The brain network parameters at the global and regional levels were calculated at each sparsity (Rubinov and Sporns, 2010; Smith et al., 2011). In this study, global parameters consisted of the clustering coefficient (Cp), characteristic path length (Lp), normalized Cp (gamma), normalized Lp (lambda), small-worldness scalar (sigma), global efficiency (Eg), and local efficiency (Eloc). Regional parameters included nodal efficiency (Ne), nodal local efficiency (NLe), and nodal clustering coefficient (NCp).

In a network, clusters (including Cp, gamma, and NCp) and Eloc measured functional segregation (Rubinov and Sporns, 2010). Paths (including Lp and lambda) and Eg measured functional integration (Achard and Bullmore, 2007; Rubinov and Sporns, 2010). Sigma, which was defined as the ratio of gamma divided by lambda, was a scalar to quantify small-worldness. The three nodal parameters (Ne, NLe, and NCp) measured the local information processing and transmission at the regional level (details in the Supplementary Material).

### Repeatability Analysis

The brain network parameters calculated according to different brain templates were not completely consistent (Wang et al., 2009; Fornito et al., 2010). The Harvard Oxford atlas (HOA) (Smith et al., 2004) with 112 brain regions and Brainnetome atlas with 246 brain regions (Fan et al., 2016) were selected to further evaluate the stability and repeatability of the results of this study.

### Statistical Analysis

SPSS software (SPSS 24.0, IBM Corporation) was used to analyze the demographic and clinical data. For the patient group, blood biochemistry and dialysis duration were analyzed using a two-sample t-test or Mann-Whitney U test. For other clinical data, a one-way analysis of variance (ANOVA) or the Kruskal-Wallis test was used to detect differences among the three groups. Categorical variables were compared using the Pearson’s χ^2^ test.

The area under the curve (AUC), as a summarized scalar, is a highly sensitive scalar independent of a single threshold selection (Lei et al., 2015; Suo et al., 2015). Therefore, a one-way analysis of covariance (ANCOVA) based on the AUC was used to determine the inter-group differences in the brain network measures. The possible confounding factors included age, sex, years of education, and SDS scores. The problem of multiple comparisons at the nodal level was addressed using the Benjamini-Hochberg false discovery rate correction method (FDR *q* < 0.05) (Genovese et al., 2002; Chen et al., 2018).

The Spearman correlation analysis was used to explore the relationship between the changed network parameters and cognitive performance in patients on MHD, with age, sex, years of education, and SDS scores as covariates.

The significance level of this study was set as 0.05, two-tailed, and *post hoc* tests (*p* < 0.05, Bonferroni corrected) were further used for pairwise comparison (Genovese et al., 2002; Chen et al., 2018).

## Results

### Demographic and Clinical Characteristics

Demographic and clinical characteristics are summarized in Table 1. MoCA and DST scores were significantly lower in patients with CI than in patients without CI and HCs (*p* < 0.05, Bonferroni corrected). The time of TMT-A in patients with CI was significantly longer than that in the other two groups (*p* < 0.05, Bonferroni corrected). SDS scores significantly increased in patients with CI, compared with HCs (*p* < 0.05, Bonferroni corrected).

**TABLE 1:** Participant demographic and clinical characteristics.

		Patients on MHD			*Post hoc* ^d^		
Variables	HCs *n* = 25	Without CI *n* = 20	With CI *n* = 19	*p*	*p1*	*p2*	*p3*
Sex (male/female)	12/13	16/4	13/6	0.076^a^	–	–	–
Age (years)	61.00 (46.00, 63.00)	50.50 (41.00, 54.00)	57.00 (51.50, 61.50)	0.068^b^	–	–	–
Education (years)	9.00 (9.00, 15.00)	12.00 (9.00, 12.00)	9.00 (9.00, 10.50)	0.088^b^	–	–	–
BMI (kg/m^2^)	23.90 (22.76, 24.50)	22.45 (20.60, 27.07)	22.77 (20.22, 23.38)	0.160^b^	–	–	–
MoCA (score)	27.00 (26.00, 28.00)	27.00 (26.00, 28.00)	24.00 (23.00, 24.50)	<0.001^b^	1.000	<0.001	<0.001
TMT-A (second)	50.69 (41.62, 59.61)	60.44 (48.02, 63.02)	87.31 (61.13, 100.45)	0.002^b^	1.000	0.002	0.038
DST-F (score)	8.00 (7.00, 9.00)	8.00 (7.00, 9.00)	7.00 (6.00, 7.00)	0.013^b^	1.000	0.044	0.019
DST-B (score)	5.00 (4.00, 6.00)	5.00 (4.00, 5.25)	4.00 (3.00, 4.00)	<0.001^b^	1.000	0.001	0.025
DST-T (score)	12.00 (10.00, 15.00)	12.50 (11.75, 14.00)	10.00 (9.00, 11.00)	0.002^b^	1.000	0.005	0.006
SDS (score)	32.00 (30.00, 38.00)	38.50 (28.75, 46.75)	44.00 (36.50, 53.00)	0.003^b^	0.549	0.002	0.153
SAS (score)	30.00 (28.00, 36.00)	36.00 (30.75, 40.25)	34.00 (31.50, 45.00)	0.071^b^	–	–	–
Hemodialysis duration (years)	–	20.00 (10.75, 88.75)	31.00 (12.00, 64.50)	0.736^c^	–	–	–
Blood urea (μmol/L)	–	12.541 ± 4.380	10.558 ± 4.851	0.188^c^	–	–	–
Blood serum creatinine (μmol/L)	–	422.85 (375.47, 621.10)	427.00 (293.45, 631.45)	0.509^c^	–	–	–
Blood uric acid (μmol/L)	–	161.65 (135.80, 201.00)	141.80 (126.70, 171.40)	0.339^c^	–	–	–
K (mmol/L)	–	3.810 ± 0.603	3.547 ± 0.620	0.188^c^	–	–	–
Ca (mmol/L)	–	1.859 ± 0.355	1.989 ± 0.263	0.202^c^	–	–	–
P (mmol/L)	–	1.08 (0.84, 1.43)	1.03 (0.72, 1.15)	0.482^c^	–	–	–
RBC (10^12^/L)	–	3.445 ± 0.639	3.726 ± 0.694	0.195^c^	–	–	–
HGB (g/L)	–	100.50 (92.75, 111.50)	110.00 (97.50, 119.00)	0.249^c^	–	–	–
HCT (g/L)	–	30.85 (27.93, 35.60)	33.60 (29.35, 38.75)	0.361^c^	–	–	–

*^a^The p-value was obtained by chi-square test.*

*^b^The p-value was obtained by the Kruskal-Wallis test.*

*^c^The p-value was obtained by two-sample t or Mann-Whitney U tests.*

*^d^Bonferroni correction (p < 0.05) was used for the post hoc test. p1 = without CI vs. HCs; p2 = with CI vs. HCs; p3 = with CI vs. without CI.*

*Continuous values are expressed as mean ± standard deviation or median [interquartile range (IQR)].*

*HCs, healthy controls; MHD, maintenance hemodialysis; CI, cognitive impairment; DST-T, Digit Span Task (total scores); DST-F, forward Digit Span Task; DST-B, backward digit span task; TMT-A, Trail Making Test A; MoCA, Montreal Cognitive Assessment Scale; SAS, self-rating anxiety scale; SDS, self-rating depression scale; K, blood potassium; Ca, blood calcium; P, blood phosphorus; RBC, red blood cell count; HGB, hemoglobin; HCT, hematocrit.*

### Alterations in Global Parameters

**FIGURE 1:**
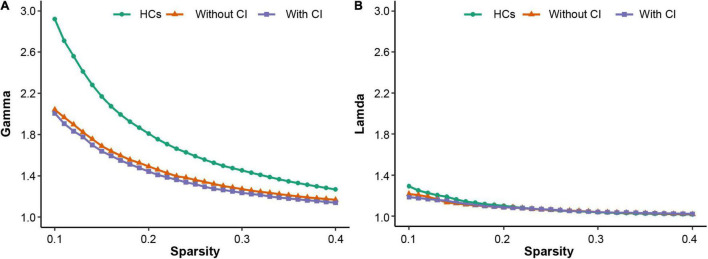
The three groups have small-worldness in a range of sparsity (0.1–0.4), i.e., gamma > 1 **(A)**, lambda ≈ 1 **(B)**. HCs, healthy controls; CI, cognitive impairment.

**FIGURE 2:**
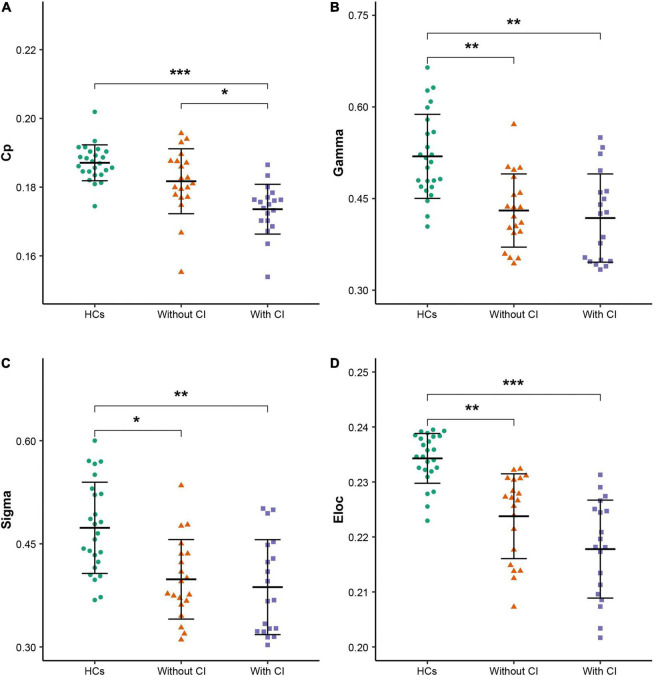
Global parameters with differences among the three groups. **(A–D)** Clustering coefficient (Cp), normalized Cp (Gamma), small-worldness scalar (Sigma), and local efficiency (Eloc), respectively. * indicates *p* < 0.05, ** indicates *p* < 0.01, *** indicates *p* < 0.001 (Bonferroni corrected). HCs, healthy controls; CI, cognitive impairment.

All participants demonstrated a small-worldness, with gamma higher than 1 and lambda approximately equal to 1 (Figure 1). According to the *post hoc* test (Table 2), sigma, gamma, and Eloc significantly decreased in both patient groups compared with those in HCs (*p* < 0.05, Bonferroni corrected). Cp significantly decreased in patients with CI compared with that in the other two groups (*p* < 0.05, Bonferroni corrected) (Figure 2).

**TABLE 2:** Area under the curve (AUC) under all thresholds at the global level.

		Patients on MHD			*Post hoc* ^b^		
Global parameters	HCs *n* = 25	Without CI *n* = 20	With CI *n* = 19	*p*	*p1*	*p2*	*p3*
Lp	0.554 ± 0.021	0.585 ± 0.041	0.591 ± 0.057	0.071^a^	–	–	–
Lambda	0.326 ± 0.009	0.323 ± 0.011	0.323 ± 0.010	0.700^a^	–	–	–
Cp	0.187 ± 0.005	0.182 ± 0.009	0.174 ± 0.00	<0.001^a^	0.076	< 0.001	0.024
Gamma	0.519 ± 0.069	0.430 ± 0.060	0.418 ± 0.072	<0.001^a^	0.003	0.001	1.000
Sigma	0.473 ± 0.066	0.398 ± 0.058	0.387 ± 0.069	0.001^a^	0.010	0.002	1.000
Eg	0.168 ± 0.005	0.161 ± 0.009	0.159 ± 0.013	0.064^a^	–	–	–
Eloc	0.234 ± 0.005	0.224 ± 0.008	0.218 ± 0.009	<0.001^a^	0.001	< 0.001	0.11

*^a^The p-value was obtained by ANCOVA with age, sex, education years, and SDS scores as covariates.*

*^b^Bonferroni correction (p < 0.05) was used for the post hoc test. p1 = without CI vs. HCs; p2 = with CI vs. HCs; p3 = with CI vs. without CI.*

*Continuous values are expressed as mean ± standard deviation.*

*HCs, healthy controls; MHD, maintenance hemodialysis; CI, cognitive impairment; Lp, characteristic path length; Lambda, normalized Lp; Cp, clustering coefficient; Gamma, normalized Cp; Sigma, small-worldness scalar; Eg, global efficiency; Eloc, local efficiency.*

### Alterations in Regional Parameters

**FIGURE 3:**
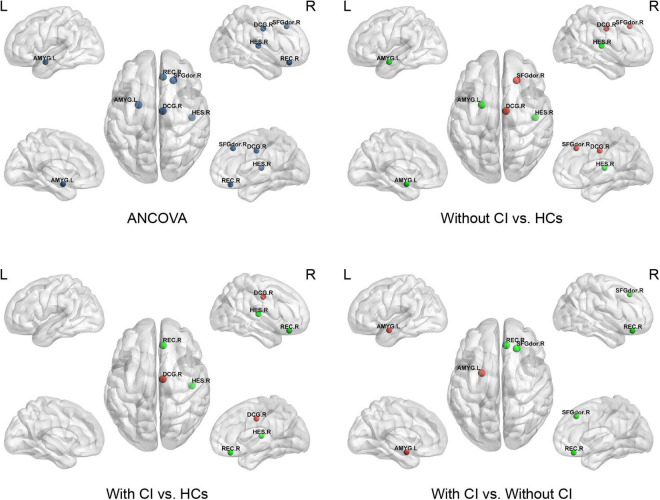
This figure shows significant differences in nodal local efficiency (NLe) among the three groups. Dark blue indicates the nodes with differences among the three groups using ANCOVA. Red represents a significant increase and green represents a significant decrease (*p* < 0.05, Bonferroni corrected). SFGdor, superior frontal gyrus dorsolateral; REC, gyrus rectus; DCG, median cingulate and paracingulate gyri; AMYG, amygdala; HES, Heschl’s gyrus.

**FIGURE 4:**
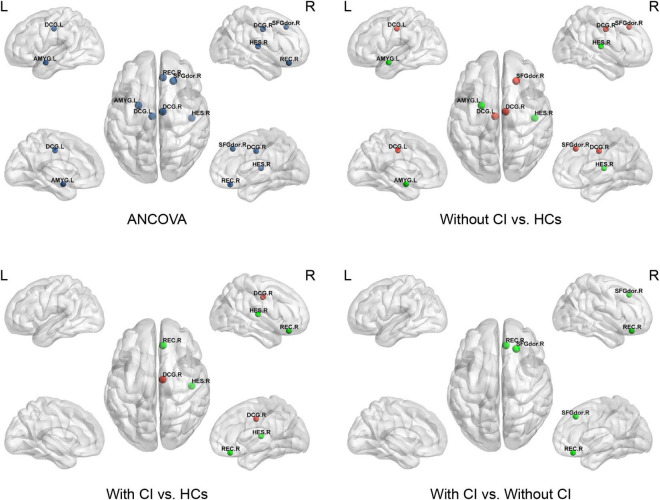
This figure shows significant differences in the nodal clustering coefficient (NCp) among the three groups. Dark blue indicates the nodes with differences among the three groups using ANCOVA. Red represents a significant increase and green represents a significant decrease (*p* < 0.05, Bonferroni corrected). SFGdor, superior frontal gyrus dorsolateral; REC, gyrus rectus; DCG, median cingulate and paracingulate gyri; AMYG, amygdala; HES, Heschl’s gyrus.

Analysis of covariances revealed that there was no significant difference in Ne among the three groups (FDR *q* > 0.05); however, several brain regions with significant alterations in NLe and NCp were observed in the frontal and temporal lobes (FDR *q* < 0.05) (Table 3 and Figures 3, 4).

**TABLE 3:** Brain regions with significant group differences in regional characteristics across the three groups.

				Patients on MHD			*Post hoc* ^b^		
AAL no.	Brain regions	Anatomical classification	HCs *n* = 25	Without CI *n* = 20	With CI *n* = 19	*p*	*p1*	*p2*	*p2*
**Nodal local efficiency**									
4	SFGdor.R	Frontal	0.231 ± 0.015	0.250 ± 0.020	0.231 ± 0.013	<0.00^a^	0.002	1.000	<0.001
28	REC.R	Frontal	0.234 ± 0.038	0.240 ± 0.038	0.167 ± 0.092	0.002^a^	1.000	0.008	0.002
34	DCG.R	Frontal	0.223 ± 0.009	0.241 ± 0.011	0.235 ± 0.013	<0.001^a^	<0.001	0.011	0.721
41	AMYG.L	Temporal	0.239 ± 0.039	0.150 ± 0.088	0.211 ± 0.069	<0.001^a^	<0.001	0.212	<0.001
80	HES.R	Temporal	0.267 ± 0.028	0.185 ± 0.095	0.138 ± 0.118	<0.001^a^	0.008	<0.001	0.327
**Nodal clustering coefficient**									
4	SFGdor.R	Frontal	0.170 ± 0.021	0.203 ± 0.038	0.170 ± 0.021	<0.001^a^	0.002	1.000	< 0.001
28	REC.R	Frontal	0.191 ± 0.042	0.206 ± 0.044	0.135 ± 0.077	<0.001^a^	1.000	0.008	0.002
33	DCG.L	Frontal	0.153 ± 0.017	0.180 ± 0.028	0.164 ± 0.018	<0.001^a^	0.002	0.088	0.637
34	DCG.R	Frontal	0.153 ± 0.015	0.184 ± 0.022	0.174 ± 0.025	0.004^a^	<0.001	0.014	0.922
41	AMYG.L	Temporal	0.203 ± 0.042	0.126 ± 0.076	0.182 ± 0.062	<0.001^a^	0.001	0.432	0.081
80	HES.R	Temporal	0.242 ± 0.039	0.167 ± 0.086	0.118 ± 0.101	<0.001^a^	0.009	< 0.001	0.193

*^a^The p-value was obtained by ANCOVA with age, sex, education years, and SDS scores as covariates and all p values corrected by FDR were less than 0.05.*

*^b^Bonferroni corrected (p < 0.05) was used for the post hoc test. p1 = without CI vs. HCs; p2 = with CI vs. HCs; p3 = with CI vs. without CI.*

*Continuous values are expressed as mean ± standard deviation.*

*HCs, healthy controls; MHD, maintenance hemodialysis; CI, cognitive impairment; SFGdor, superior frontal gyrus, dorsolateral; REC, gyrus rectus; DCG, median cingulate and paracingulate gyri; AMYG, amygdala; HES, Heschl gyrus.*

Compared with HCs, regional parameters of the right superior frontal gyrus, dorsolateral (SFGdor), and bilateral median cingulate and paracingulate gyri (DCG) increased significantly in patients without CI, and significantly decreased in the left amygdala (AMYG) and right Heschel (HES) (*p* < 0.05, Bonferroni corrected).

Compared with HCs, the regional parameters of the right DCG were significantly increased in patients with CI, while the right gyrus rectus (REC) and HES were significantly decreased (*p* < 0.05, Bonferroni corrected).

Relative to patients without CI, significantly increased and decreased nodal parameters were located in the left AMYG and right frontal lobes (right SFGdor and REC), respectively (*p* < 0.05, Bonferroni corrected).

### Correlation Analysis

The detailed results of the correlation analysis are shown in Table 4. Cp was associated with the MoCA score (*r* = 0.505; FDR *q* = 0.004). For the right SFGdor, the MoCA score was positively correlated with the NLe (*r* = 0.403; FDR *q* = 0.03) and NCp (*r* = 0.425; FDR *q* = 0.021). For the right REC, the MoCA, DST-B, and DST-T scores were positively associated with the NLe (*r* = 0.4, FDR *q* = 0.03; *r* = 0.494, FDR *q* = 0.005; and *r* = 0.49, FDR *q* = 0.01, respectively) and NCp (*r* = 0.448, FDR *q* = 0.021; *r* = 0.485, FDR *q* = 0.012; and *r* = 0.446, FDR *q* = 0.024, respectively). For the left AMYG, the MoCA score was negatively correlated with the NCp (*r* = –0.378; FDR *q* = 0.036), and the TMT-A time was positively correlated with both the NLe (*r* = 0.417; FDR *q* = 0.04) and NCp (*r* = 0.477; FDR *q* = 0.012).

**TABLE 4:** Correlation analysis of changed brain network parameters and cognitive function in patients on MHD.

	MoCA			TMT-A			DST-F			DST-B			DST-T		
	*r*	*p*	FDR *q*	*r*	*p*	FDR *q*	*r*	*p*	FDR *q*	*r*	*p*	FDR *q*	*r*	*p*	FDR *q*
**Global parameters**															
Cp	0.505	0.001	**0.004**	−0.321	0.047	0.116	0.037	0.825	0.825	0.121	0.464	0.501	0.124	0.452	0.452
Gamma	0.089	0.588	0.682	−0.088	0.592	0.677	0.166	0.311	0.415	0.111	0.501	0.501	0.142	0.388	0.452
Sigma	0.068	0.682	0.682	−0.069	0.677	0.677	0.176	0.283	0.415	0.161	0.327	0.501	0.18	0.273	0.452
Eloc	0.314	0.052	0.104	−0.306	0.058	0.116	0.198	0.227	0.415	0.205	0.211	0.501	0.245	0.133	0.452
**Nodal local efficiency**															
SFGdor.R	0.403	0.011	**0.03**	−0.31	0.055	0.138	0.191	0.243	0.304	−0.053	0.746	0.746	0.112	0.498	0.623
REC.R	0.4	0.012	**0.03**	−0.271	0.095	0.158	0.344	0.032	0.16	0.494	0.001	**0.005**	0.49	0.002	**0.01**
DCG.R	0.221	0.176	0.22	0.011	0.948	0.948	0.226	0.166	0.277	0.227	0.164	0.41	0.262	0.107	0.269
AMYG.L	−0.336	0.037	0.062	0.417	0.008	**0.04**	−0.24	0.142	0.277	−0.109	0.509	0.636	−0.2	0.223	0.372
HES.R	0.15	0.363	0.363	−0.135	0.413	0.516	−0.068	0.681	0.681	0.146	0.376	0.627	0.047	0.778	0.778
**Nodal clustering coefficient**															
SFGdor.R	0.425	0.007	**0.021**	−0.351	0.028	0.084	0.179	0.276	0.414	0.006	0.973	0.973	0.134	0.416	0.499
REC.R	0.448	0.004	**0.021**	−0.247	0.13	0.26	0.277	0.088	0.264	0.485	0.002	**0.012**	0.446	0.004	**0.024**
DCG.L	0.317	0.05	0.075	0.008	0.96	0.96	0.075	0.652	0.782	0.149	0.366	0.457	0.163	0.322	0.483
DCG.R	0.212	0.194	0.219	0.025	0.879	0.96	0.219	0.181	0.362	0.25	0.125	0.375	0.261	0.109	0.218
AMYG.L	−0.378	0.018	**0.036**	0.477	0.002	**0.012**	−0.302	0.062	0.264	−0.175	0.288	0.457	−0.276	0.089	0.218
HES.R	0.202	0.219	0.219	−0.162	0.323	0.485	−0.045	0.785	0.785	0.144	0.381	0.457	0.063	0.702	0.702

*Cp, clustering coefficient; Gamma, normalized Cp; Sigma, small-worldness scalar; Eloc, local efficiency; SFGdor, superior frontal gyrus dorsolateral; REC, gyrus rectus; DCG, median cingulate and paracingulate gyri; AMYG, amygdala; HES, Heschl gyrus; MoCA, Montreal Cognitive Assessment Scale; DST-T, Digit Span Task (total scores); DST-F, forward Digit Span Task; DST-B, backward Digit Span Task; TMT-A, Trail Making Test A. The bold values indicate that p values corrected by FDR were less than 0.05.*

### Results of Repeatability

The global parameters using the HOA and Brainnetome atlas were similar to the results from AAL templates (Supplementary Table 1). According to the HOA and Brainnetome atlas, significant changes in nodal parameters were not only found in the frontal and temporal lobes but were also found in other brain regions, such as the insula and thalamus (Supplementary Table 2).

## Discussion

This study, based on rs-fMRI, revealed abnormal changes in functional brain networks in patients on MHD by using graph theory analysis, and the main findings are as follows: (1) in comparison with HCs, global properties, including sigma, gamma, and Eloc, were significantly decreased in patients with and without CI. Cp was significantly lower in patients with CI than in the other two groups; (2) in comparison with HCs, the altered nodal parameters (NCp and NLe) of patients on MHD were mainly located in the frontal and temporal lobes; (3) in comparison with patients without CI, the nodal parameters (NCp and NLe) of the prefrontal cortex (SFGdor and REC) were significantly decreased and NLe of the amygdala was significantly increased in patients with CI; and (4) several altered global and regional parameters of patients on MHD were significantly correlated with cognition scores. These results contribute to the understanding of the underlying neuropathological mechanism of cognitive decline in patients on MHD.

### Altered Global Parameters

The human brain network is a network model with small-worldness (Zhang et al., 2011; Suo et al., 2015), which combines a higher Cp and shorter Lp to satisfy the opposing demands of global and local processing and ensure highly efficient transformation and processing of information at the global and regional levels (Kaiser and Hilgetag, 2006; Bullmore and Sporns, 2009). The more the brain network structure deviates from the optimal state, the more prone it is to abnormal brain function (Rubinov and Sporns, 2010; Xu et al., 2019). The results of this study demonstrated that the three groups had small-worldness (Figure 1); however, the small-worldness scalar (sigma) of both patient group was significantly lower than that of HCs. This reflects the fact that the overall structure of the small-world network does not reach the optimal state, which further affects the transmission and processing of whole brain information. A study (Wu et al., 2020) revealed that compared with HCs, the sigma of patients on hemodialysis was also significantly reduced, but the reduced Lp and lambda in patients was not consistent with our results. The cognitive function of patients with ESRD is affected by dialysis duration (Brouhard et al., 2000), and the brain network architecture is related to cognitive level (Liu et al., 2012). Given that the duration of hemodialysis among patients with ESRD in this study was longer than that in previous studies (Wu et al., 2020; Yue et al., 2021), it is postulated that these inconsistent results may be due to hemodialysis duration. According to the definition of Eloc and gamma (Sporns and Zwi, 2004; Rubinov and Sporns, 2010), their decreased values in the patient group reflect the decreased information transmission ability of patients on MHD at the regional level. In addition, these results further confirm that hemodialysis can adversely affect the brain networks of patients (Chou et al., 2019; Wu et al., 2020).

At the global level, an important finding was that the Cp of patients with CI decreased significantly compared with patients without CI (*p* < 0.05, Bonferroni corrected), which implies that the disruption of the brain network was more serious in patients with CI. Cp measures the extent of local cliquishness of the network, i.e., functional segregation (Rubinov and Sporns, 2010). A decrease in the Cp reflects a decrease in the information transmission efficiency at the regional level. In this study, patients with CI had significantly reduced Cp compared with patients without CI, which implies local information processing was significantly reduced in those patients. Previous studies (Park et al., 2020; Xiong et al., 2020) have confirmed a close relationship between Cp and cognitive function. Moreover, the correlation analysis in this study revealed Cp was significantly and positively correlated with the global cognitive function (MoCA) in patients on MHD. Therefore, the reduction of local information processing may be an important neuropathological mechanism for the decline of global cognitive function in patients on MHD.

### Altered Regional Parameters

Consistent with the changes of the Cp and Eloc, NCp, and NLe were the significantly altered nodal parameters. They mainly affected the frontal and temporal lobes. The frontal and temporal lobes play an important role in several cognitive functions, such as executive function, attention, and memory (Chayer and Freedman, 2001; Oh and Jagust, 2013; Fang et al., 2016). Consequently, the abnormal node attributes in the frontal-temporal lobe may be associated with potential cognitive decline in patients on MHD.

Further analysis found that, except for the amygdala, the nodal parameters of the frontal and temporal cortices in patients with CI were significantly or slightly lower than those in patients without CI. This indicated that the more severe brain network damage in patients on MHD with CI mainly occurred in the frontal-temporal lobe, particularly in the prefrontal lobe [NCp and NLe of the prefrontal lobe in patients with CI were significantly lower than those in patients without CI (*p* < 0.05, Bonferroni corrected)]. A study confirmed that the impairment of the brain network in the prefrontal lobe in patients with ESRD with CI was more serious than in patients without CI (Yue et al., 2021). However, unlike in the previous study, patients on MHD with CI in our study did not show a significant reduction in node attributes in the precuneus and posterior cingulate gyrus. This may have been due to the use of different nodal parameters for evaluation of the changes in node attributes; a more important reason being the different hemodialysis duration, which was similar to the difference at the global level. In addition, compared with HCs, the nodal parameters of the frontal lobe increased and those of the temporal lobe decreased in patients on MHD without CI. This result is similar to the conclusion of a study by Wu et al. (2020) that suggested that the nodal degree of the frontal cortex in patients on hemodialysis is higher than that in HCs, and the nodal degree of the temporal lobe is lower than that in HCs. The increase in degree centrality in multiple brain regions, including the middle frontal gyrus, may be an important compensatory mechanism for patients with Alzheimer’s disease to delay dementia (Behfar et al., 2020). Similarly, the enhancement of frontal cortex activity is also a compensatory mechanism for healthy elderly people to resist neurodegeneration and maintain normal cognitive levels (Scheller et al., 2018). Therefore, the increased nodal parameters of the frontal lobes in patients on MHD without CI in this study may reflect an adaptive compensatory mechanism of delaying cognitive decline in patients on MHD. Taken together, these results suggest that the occurrence of CI in patients on MHD may be related to the impairment of the frontotemporal brain network and the weakening of frontal nerve compensation.

The nodal parameters of the amygdala (including NCp and NLe) in patients on MHD without CI were significantly lower than those in HCs, which is consistent with the results of the previous studies (Wu et al., 2020; Yue et al., 2021). The amygdala is an important structure for the regulation and processing of emotions (Dalton et al., 2005). Decreased functional connectivity between the amygdala and multiple brain regions may be related to depression in patients with ESRD (Chen et al., 2017). However, further analysis revealed that the NLe of the amygdala in patients with CI was significantly higher than that in patients without CI, even if SDS scores were taken as a covariate. This indicated that abnormal brain connectivity in the amygdala of patients with CI may not be related to abnormal emotion regulation only. We speculate that the abnormal brain connectivity of the amygdala in patients with CI may be the result of interaction between cognition and emotion regulation since there is an interaction effect between emotion and cognitive function (Brosch et al., 2013).

### Relationship Between Altered Network Parameter and Cognitive Function in Patients on Maintenance Hemodialysis

Clustering coefficient was significantly and positively correlated with the MoCA score. Although more notably, the nodal parameters of the brain regions (right SFGdor and REC, and left AMYG), with significant differences among the patient groups, were significantly correlated with the cognitive performance of patients on MHD. Specifically, both the NCp and NLe of the prefrontal lobe (right SFGdor and REC) were significantly and positively correlated with the MoCA score. In patients with ESRD, the prefrontal lobe is an important brain region related to cognition, and functional and structural abnormalities of the prefrontal lobe have been observed in previous studies (Ma et al., 2015; Jin et al., 2020). Other studies have confirmed that for patients with ESRD, prefrontal connectivity is significantly correlated with global cognitive function (i.e., the MoCA score) (Wu et al., 2020; Huang et al., 2021). These results suggest that weakening of local information transmission, especially in the prefrontal lobe, may play an important role in the global cognitive decline of patients on MHD and may become an imaging marker for early evaluation of cognitive decline in such patients. Additionally, there was a significant positive correlation between the nodal parameters (NCp and NLe) of the right REC and short-term memory function scores (DST-B and DST-T scores). The REC plays an important role in memory function; however, both memory and language function would be negatively affected should the REC be resected (Joo et al., 2016). The REC is also an important part of the orbitofrontal cortex, which is directly involved in memory processing (Petrides, 2007). Accordingly, the functional disconnect of the REC may be involved, at least in part, in the disruption of short-term memory function in patients on MHD. The nodal parameters of the left AMYG were negatively correlated with the MoCA score and positively correlated with TMT-A time (a test for executive function). The AMYG can participate in and coordinate executive attention through the amygdala–prefrontal circuit (Salzman and Fusi, 2010). Consequently, the abnormal node attribute of the AMYG in this study may be related to cognitive deficits in patients on MHD, especially executive function.

### Repeatability Analysis

The global and regional attributes of the three different anatomical atlases have high similarity, which indicates that the results of this study have good repeatability and stability. Particularly at the global level, ANCOVA results showed that the three atlases obtained more consistent results.

### Limitations

This study had some limitations. First, the sample size of this study was small. The cross-sectional study design of this study may make it difficult to reveal the longitudinal changes in functional brain networks and cognitive function in patients on MHD. This needs to be further verified by increasing the sample size and conducting longitudinal studies in the future. Second, the evaluation of cognitive function in this study lacks a systematic approach and objectivity, which may have resulted in some bias. Third, the lack of detailed grouping in this study to distinguish the primary and secondary diseases of ESRD may also affect the results.

### Conclusion

This study confirmed that the topological architecture of the functional brain network in patients on MHD was disrupted. Furthermore, the impairment of the brain network in patients with CI was more severe than that in patients without CI, particularly in the prefrontal cortex. Changes in brain network parameters at global and regional levels are related to the cognitive function of patients on MHD. Functional brain network analysis based on graph theory can provide an important imaging basis for the neuropathological mechanism of cognitive dysfunction in patients on MHD.

## Data Availability Statement

The data analyzed in this study is subject to the following licenses/restrictions: Raw data will not be made public to protect the subjects’ privacy. Requests to access these datasets should be directed to JW, cjr.wujianlin@vip.163.com.

## Ethics Statement

The studies involving human participants were reviewed and approved by Ethics Committee of the Affiliated Zhongshan Hospital of Dalian University. The patients/participants provided their written informed consent to participate in this study.

## Author Contributions

JW and DZ contributed to the conception and design of the study. DZ, YC, and LL performed the statistical analysis and wrote the manuscript. HW, QX, CC, and LJ performed the experiments. All authors contributed to manuscript revision, read, and approved the submitted version.

## Conflict of Interest

The authors declare that the research was conducted in the absence of any commercial or financial relationships that could be construed as a potential conflict of interest.

## Publisher’s Note

All claims expressed in this article are solely those of the authors and do not necessarily represent those of their affiliated organizations, or those of the publisher, the editors and the reviewers. Any product that may be evaluated in this article, or claim that may be made by its manufacturer, is not guaranteed or endorsed by the publisher.

## Footnotes

1
http://rfmri.org/DPABI

2
www.mathworks.com

3
http://www.nitrc.org/projects/gretna/
