# Supplementary material for: Associations of the Disrupted Functional Brain Network and Cognitive Function in End-Stage Renal Disease Patients on Maintenance Hemodialysis: A Graph Theory-Based Study of Resting-State Functional Magnetic Resonance Imaging
Source: Front Hum Neurosci. 2021 Dec 13;15:716719. doi: 10.3389/fnhum.2021.716719 (PMC8710547; doi:10.3389/fnhum.2021.716719)
Supplement: Supplementary file 1 [file Data_Sheet_1.pdf]

Supplementary Material

Part 1 Global parameters

Small-world related parameters

(1) Clustering coefficient (Cp): The clustering coefficient of node *i* (NCp_i) refers to the actual number of edges among all the first neighbor nodes of node *i* divided by the maximum possible number of edges. The clustering coefficient of a network (Cp) is the average of the clustering coefficients of all nodes, which quantifies the extent of the local cliquishness of the network (Watts and Strogatz, 1998).

(2) Characteristic path length (Lp): The average shortest path length of node *i* refers to the average value of the shortest path length between node *i* and all other nodes. The characteristic path length (Lp) is equal to the average value of the average shortest path length of all nodes in the network, which measures the extent of the average connectivity or overall efficiency of the network(Watts and Strogatz, 1998).

(3) Small-worldness scalar (sigma): To quantify small-worldness, it is necessary to compare the Cp and Lp of the actual network with those of the random network to obtain the standardized Cp (gamma) and standardized Lp (lambda) (Maslov and Sneppen, 2002). Sigma is then calculated, and sigma = gamma/ lambda. Gamma > 1 and lambda ≈ 1 (i.e., sigma > 1) indicates that the network has a small-worldness (Watts and Strogatz, 1998) .

Efficiency related parameters

(1) Global efficiency (Eg): Eg refers to the average value of the reciprocal of the shortest path length between each pair of nodes, which measures the efficiency of information transmission at the global level of the network (Maslov and Sneppen, 2002).

(2) Local efficiency (Eloc): Eloc refers to the average value of the nodal local efficiency (NLe) of all nodes, which measures the information integration ability at the local level.

Part 2 Regional parameters

To evaluate the change in the local functional network structure, this study selects the following parameters to measure the local-level information transmission capacity:

(1) Nodal efficiency (Ne): Ne refers to the average value of the reciprocal of the shortest path length between node *i* and all other nodes, which can measure the information dissemination ability of node *i* and other nodes in the network.

(2) Nodal local efficiency (NLe): NLe measures the ability of information exchange between the first neighbor nodes of node *i*.

(3) Nodal clustering coefficient (NCp): The clustering coefficient of node *i* (NCp_i) refers to the actual number of edges among all the first neighboring nodes of node *i* divided by the maximum possible number of edges.

Part 3 Repeatability analysis

Supplementary Table 1 Area under the curve (AUC) under all thresholds at the global level from Harvard-Oxford Atlas and Brainnetome Atlas

		Patients on MHD			Post hoc[b]		
Global parameters	HCs<br>n = 25	Without CI<br>n = 20	With CI<br>n = 19	*p*	*p1*	*p2*	*p3*
Harvard-Oxford Atlas							
Lp	0.460 ± 0.064	0.394 ± 0.059	0.383 ± 0.066	0.198[a]	-	-	-
Lambda	0.546 ± 0.022	0.578 ± 0.048	0.580 ± 0.072	0.346[a]	-	-	-
Cp	0.323 ± 0.007	0.321 ± 0.009	0.319 ± 0.007	0.007[a]	0.354	0.005	0.232
Gamma	0.498 ± 0.065	0.422 ± 0.061	0.408 ± 0.069	0.002[a]	0.013	0.004	1.000
Sigma	0.186 ± 0.004	0.181 ± 0.013	0.174 ± 0.013	0.008[a]	0.034	0.014	1.000
Eg	0.170 ± 0.005	0.162 ± 0.011	0.162 ± 0.015	0.239[a]	-	-	-
Eloc	0.234 ± 0.004	0.225 ± 0.008	0.220 ± 0.010	< 0.001[a]	0.008	< 0.001	0.269
Brainnetome Atlas							
Lp	0.469 ± 0.060	0.392 ± 0.055	0.394 ± 0.066	0.225[a]			
Lambda	0.521 ± 0.011	0.547 ± 0.039	0.543 ± 0.053	0.305[a]	-	-	-
Cp	0.316 ± 0.004	0.315 ± 0.006	0.313 ± 0.005	0.012[a]	0.237	0.010	0.534
Gamma	0.497 ± 0.062	0.412 ± 0.055	0.412 ± 0.067	0.001[a]	0.003	0.002	1.000
Sigma	0.186 ± 0.007	0.181 ± 0.013	0.173 ± 0.015	0.002[a]	0.007	0.006	1.000
Eg	0.176 ± 0.003	0.169 ± 0.010	0.171 ± 0.013	0.250[a]	-	-	-
Eloc	0.240 ± 0.003	0.230 ± 0.007	0.227 ± 0.007	< 0.001[a]	<0.001	<0.001	1.000

[a]The p-value was obtained by ANCOVA with age, sex, years of education, and SDS scores as covariates.
[b]Bonferroni correction ($p < 0.05$) was used for the post hoc test. *p1* = without CI vs. HCs; *p2* = with CI vs. HCs; *p3* = with CI vs. without CI
Continuous values are expressed as mean ± standard deviation
HC, healthy controls; MHD, maintenance hemodialysis; CI, cognitive impairment; Lp, characteristic path length; Lambda, normalized Lp; Cp, clustering coefficient; Gamma, normalized Cp; Sigma, small-worldness scalar; Eg, global efficiency; Eloc, local efficiency.

Supplementary Table 2 Brain regions with significant group differences in regional characteristics across different brain templates.

				Patients on MHD			Post hoc[b]		
Nodal parameters	Brain regions	Anatomical classification	HCs n = 25	Without CI n = 20	With CI n = 19	*p*	*p1*	*p2*	*p3*
Harvard-Oxford Atlas									
NCp	T3a.L	Temporal	0.215 ± 0.043	0.131 ± 0.081	0.157 ± 0.084	< 0.001[a]	< 0.001	0.009	1.000
Brainnetome Atlas									
Ne	MFG.L	Frontal	0.165 ± 0.014	0.189 ± 0.023	0.191 ± 0.017	< 0.001[a]	< 0.001	< 0.001	1.000
Ne	STG.R	Frontal	0.203 ± 0.018	0.179 ± 0.021	0.180 ± 0.022	< 0.001[a]	< 0.001	0.002	1.000
NLe	Amyg.L	Frontal	0.237 ± 0.040	0.137 ± 0.095	0.166 ± 0.075	< 0.001[a]	< 0.001	0.003	1.000
NCp	INS.R	Insular	0.217 ± 0.031	0.190 ± 0.055	0.131 ± 0.067	< 0.001[a]	0.188	< 0.001	0.012
NCp	Amyg.L	Temporal	0.192 ± 0.040	0.115 ± 0.080	0.131 ± 0.060	< 0.001[a]	< 0.001	0.003	1.000
NCp	Tha.R	Subcortical	0.210 ± 0.041	0.146 ± 0.070	0.141 ± 0.056	< 0.001[a]	< 0.001	< 0.001	1.000

[a]The p-value was obtained by ANCOVA with age, sex, years of education, and SDS scores as covariates.
[b]Bonferroni correction ($p < 0.05$) was used for the post hoc test. *p1* = without CI vs. HCs; *p2* = with CI vs. HCs; *p3* = with CI vs. without CI
Continuous values are expressed as mean ± standard deviation
HCs, healthy controls; MHD, maintenance hemodialysis; CI, cognitive impairment; Ne, nodal efficiency; NLe, nodal clustering coefficient; NCp, nodal clustering coefficient; T3a.L, inferior temporal gyrus; MFG, middle frontal gyrus; STG, superior temporal gyrus; Amyg, amygdala; INS, insular gyrus; Tha, thalamus.

References

[1] Watts, D. J., and Strogatz, S. H. (1998). Collective dynamics of “small-world” networks. Nature. 393(6684), 440-442. doi: 10.1038/30918.

[2] Maslov, S., and Sneppen, K. (2002). Specificity and stability in topology of protein networks. Science. 296(5569), 910-913. doi: 10.1126/science.1065103.
